# [^18^F]FDG PET/CT in Short-Term Complications of COVID-19: Metabolic Markers of Persistent Inflammation and Impaired Respiratory Function

**DOI:** 10.3390/diagnostics12040835

**Published:** 2022-03-29

**Authors:** Eva María Triviño-Ibáñez, Beatriz María Jiménez-Rodríguez, Teodoro Rudolphi-Solero, Encarnación Yolanda García-Rivero, Antonio Rodríguez-Fernández, José Manuel Llamas-Elvira, Manuel Gómez-Río, Concepción Morales-García

**Affiliations:** 1 Department of Nuclear Medicine, Virgen de las Nieves University Hospital, 18014 Granada, Spain; eva_gor@hotmail.com (E.M.T.-I.); encarnacion.garcia.rivero.sspa@juntadeandalucia.es (E.Y.G.-R.); antonio.rodriguez.fernandez.sspa@juntadeandalucia.es (A.R.-F.); josem.llamas.sspa@juntadeandalucia.es (J.M.L.-E.); manuel.gomez.rio.sspa@juntadeandalucia.es (M.G.-R.); 2 Biosanitary Research Institute of Granada-IBS, 18012 Granada, Spain; beajirod@gmail.com (B.M.J.-R.); concepcion.morales.sspa@juntadeandalucia.es (C.M.-G.); 3 PhD P in Clinical Medicine and Public Health, Department of Microbiology, School of Medicine, University of Granada, 18011 Granada, Spain; 4 Department of Pneumology, Virgen de las Nieves University Hospital, 18014 Granada, Spain

**Keywords:** COVID-19, [^18^F]FDG-PET/CT, respiratory function test, inflammatory, complications, SARS-CoV-2

## Abstract

SARS-CoV-2 virus infects organs other than the lung, such as mediastinal lymph nodes, spleen, and liver, but, to date, metabolic imaging studies obtained in short-term follow-ups of patients hospitalized with severe COVID-19 infection are rare. Our objective was to evaluate the usefulness of [^18^F]FDG-PET/CT in the short-term follow-up of patients admitted for COVID-19 pneumonia and to explore the association of the findings with clinical prognostic markers. The prospective study included 20 patients with COVID-19 pneumonia (November 2020–March 2021). Clinical and laboratory test findings were gathered at admission, 48–72 h post-admission, and 2–3 months post-discharge, when [^18^F]FDG-PET/CT and respiratory function tests were performed. Lung volumes, spirometry, lung diffusion capacity for carbon monoxide (DLCO), and respiratory muscle strength were measured. Volumetric [^18^F]FDG-PET/CT results were correlated with laboratory and respiratory parameters. Eleven [^18^F]FDG-PET/CT (55%) were positive, with hypermetabolic mediastinal lymphadenopathy in 90.9%. Mediastinal lesion’s SUVpeak was correlated with white cells’ count. Eleven (55%) patients had impaired respiratory function, including reduced DLCO (35%). SUVpeak was correlated with %predicted-DLCO. TLG was negatively correlated with %predicted-DLCO and TLC. In the short-term follow-up of patients hospitalized for COVID-19 pneumonia, [^18^F]FDG-PET/CT findings revealed significant detectable inflammation in lungs and mediastinal lymph nodes that correlated with pulmonary function impairment in more than half of the patients.

## 1. Introduction

There is growing interest in the diagnosis, prognosis, and optimal clinical management of the sequelae of acute COVID-19 infection.

In the acute phase of infection, the epidemiology, clinical characteristics, results of standard clinical laboratory tests, lung CT appearance, treatment strategies, and outcomes in patients with COVID-19 have been reported in previous studies [1]. Imaging techniques, especially high-resolution computed tomography (HRCT), have demonstrated a relevant diagnostic role [2], and multiple studies have been published on radiological findings in patients with COVID-19 pneumonia, especially during the acute phase and, more recently, over the short and medium terms [2,3].

The SARS-CoV-2 virus has been shown to infect organs other than the lung, such as the mediastinal lymph nodes, spleen, and liver, quantitative case studies in patients with COVID-19 are rare [3,4]. Such information can be obtained through the use of [^18^F]- 2-Fluoro-2-Deoxy-Glucose ([^18^F]FDG) positron emission tomography/computed tomography (PET/CT), which is commonly used to assess inflammatory and infectious lung diseases [5].

The complementary functional information provided by [^18^F]FDG-PET/CT, which has been shown to be useful for diagnosing inflammatory and infectious lung diseases, estimating their severity, monitoring their evolution, and evaluating therapeutic response [4,5], can help elucidate the pathophysiological mechanisms of COVID-19. The value of [^18^F]FDG-PET/CT has been reported in patients with respiratory infections caused by other coronaviruses, such as MERS-CoV and SARS-CoV [6,7], as well as in patients with acute COVID-19 infection [4,8].

The [^18^F]FDG-PET/CT studies of asymptomatic cancer patients described the incidental detection of interstitial pneumonia compatible with possible acute SARS-CoV-2 infection [7], and researchers have begun to examine the potential role of [^18^F]FDG-PET/CT in its diagnosis and treatment [8]. As well as visual interpretation by an experienced specialist, [^18^F]FDG-PET/CT also offers a semiquantitative approach to glycemic metabolism and, therefore, the intensity of inflammatory activity. Besides the standardized uptake value (SUV), recent studies in oncology have yielded additional parameters such as the metabolic tumor volume (MTV) and total lesion glycolysis (TLG) [9], which could be used to estimate inflammatory activity in lungs or extrapulmonary organs, especially lymph nodes. Studies of noncritical hospitalized patients have highlighted the possible relevance of lymph node hypermetabolism, quantified by the maximum SUV (SUVmax) in PET images, proposing that the highest SUVmax values for lesions and lymph nodes may indicate an increased severity of the infection and may predict a poor prognosis [3,4].

With this background, we hypothesized that [^18^F]FDG-PET/CT could be useful to characterize pulmonary sequelae of COVID-19 infection. The objective of this study was to evaluate the usefulness of [^18^F]FDG-PET/CT in the short-term follow-up of patients admitted for COVID-19 pneumonia and to explore the association of findings with clinical prognostic markers

## 2. Materials and Methods

### 2.1. Patients

This prospective, longitudinal, observational study enrolled consecutive COVID-19 patients at their follow-up visit 1–2 months after discharge from a third-level hospital between 27 November 2020 to 1 March 2021.

Study inclusion criteria were confirmation of COVID-19 in accordance with WHO guidelines [10] by a positive RT-PCR result for nasopharyngeal swabs, hospital admission between November 2020 to March 2021 (dates of “third wave” in Spain), and findings of ground-glass opacity or consolidation on chest HRCT scan or X-ray at admission. Exclusion criteria were age under 18 years, absence of microbiological confirmation of COVID-19 infection, history or presence of pulmonary fibrosis, active or uncontrolled COVID-19 infection at the time of the [^18^F]FDG-PET/CT study, history or suspicion of oncological disease, pregnancy, and inability to sign informed consent.

The study was approved by the local Research Ethics Committee, and written, informed consent was obtained from all participants. Personal protective equipment was available for all staff, and COVID-19 infection prevention guidelines were always rigorously followed [11].

### 2.2. Clinical Information and Laboratory Test Results

For all patients, data were gathered from electronic medical records, including the results of clinical and laboratory tests at admission, at 48–72 h post-admission, and at the follow-up PET/CT examination. Analytical data included complete blood count, standard blood biochemistry, acute phase reactants, coagulation status [12], and neutrophil/lymphocyte ratios (NLRs). All patients underwent RT-PCR for nucleic acid testing of SARS-CoV-2.

### 2.3. Respiratory Function Tests

Respiratory function tests were performed at 2–3 months after hospital discharge. Spirometry results (in mL and % predicted) were obtained for forced vital capacity (FVC), forced expiratory volume in 1 s (FEV1), and FEV1/FVC ratio. Body plethysmography was used to measure the residual volume (RV, in mL and % predicted), and total lung capacity (TLC, in mL and % predicted). The diffusing capacity of the lungs for carbon monoxide (DLCO) and the CO transfer coefficient (KCO) were expressed in absolute numbers and as % predicted. The results of the 6-min walk test (TM6M) were expressed as distance (in m) and % oxygen saturation at start and finish. Specifically trained personnel carried out functional tests using MasterScreen Body equipment (Jaeger, Hoechberg, Germany), considering reference values for the Mediterranean population and acceptability criteria established by European and Spanish regulations [13,14].

### 2.4. PET/CT Data Acquisition

After two consecutive negative RT-PCR test results for SARS-CoV-2 nucleic acid, confirming that patients were no longer infected, patients underwent [^18^F]FDG-PET/CT imaging (Siemens Biograph Vision 600 PET/CT, Siemens Healthcare, Erlangen, Germany), always performed within 2–3 months after discharge from hospital. The test protocol was based on international recommendations [15]. Patients were administered intravenously with the radiopharmaceutical (3.7–4.81 MBq/kg) at rest after fasting for at least 6 h with adequate hydration as long as their capillary blood glucose level was below 6.8 mmol/L. Image acquisition (whole body in 3D) started at 50–60 min post-injection with the acquisition of a topogram (50 mA, 120 kV), followed by helical CT without contrast (170 mA, 120 kV) and the acquisition of PET images with coverage from skull base to mid-thigh.

### 2.5. PET/CT Image Interpretation

The [^18^F]FDG-PET/CT and chest CT images were independently analyzed by two nuclear medicine physicians (E.M.T.I. and M.G.M.) with a great deal of experience in the interpretation of cardiothoracic images, using syngo.via version VB40B software (Siemens Healthcare, Erlangen, Germany). They were blinded to the biological and clinical data of patients. Discrepancies in interpretations were resolved by consensus with a third expert nuclear medicine physician (A.R.F.).

The [^18^F]PET/CT data were transferred to a computer workstation (syngo.via) for the co-registration of PET and CT images. Regions of interest (ROIs) were drawn on CT images of lungs around areas with evident loss of aeration and adjacent areas of normal appearance. ROIs were also drawn on CT images of mediastinal lymph nodes. The ROIs drawn on the CT images of each patient were transferred to the co-registered PET images and the amount of [^18^F]FDG pathological uptake was calculated for each ROI, determining maximum, peak, and minimum SUVs, normalized by body weight (SUVmax, SUVpeak, and SUVmin, respectively) and lean body mass (SUL); metabolic tumoral volume (MTV; volume of pixels in the ROI with SUVmax >40%); and total lesion glycolysis (TLG; MTV multiplied by SUVmean).

### 2.6. Chest CT and X-ray Image Interpretation

Upon their diagnosis, all patients underwent chest X-ray in posterior-anterior and lateral projections, reported by specialist radiologists according to current recommendations [16,17]. They characterized the density (alveolar, ground glass, or mixed), distribution (central, peripheral, or diffuse), location (unilateral or bilateral), and extent (unilobar or multilobar).

### 2.7. Statistical Analysis

All measurements for each participant were independently conducted by two nuclear medicine physicians, considering the mean value in statistical analyses. Absolute numbers and percentages were calculated for categorical variables and means with standard deviation (SD) for continuous variables. For comparisons of quantitative data between the positive and negative PET groups, the Student’s *t*-test was applied when the distribution was normal and the Mann–Whitney U test when it was not. Associations with categorical variables were evaluated by constructing contingency tables, applying the chi-square test for individual comparisons and Fisher’s exact test for multiple comparisons. Volumetric [^18^F]FDG-PET/CT results were correlated with laboratory test results and respiratory function parameters by using Spearman’s rank correlation coefficient. IBM SPSS version 15.0 (IBM Corp, Armonk, NY, USA) and R software were used for statistical analyses. A *p* ≤ 0.05 was considered significant in all tests.

## 3. Results

The study included 20 patients (60% males) with a mean age of 55.85 ± 9.28 years admitted for pneumonia and/or respiratory failure between 27 November 2020 and 1 March 2021 (during the “third wave” of COVID-19 in Spain). The mean hospital stay was 16.70 ± 11.99 days. Table 1 summarizes the baseline characteristics of the patients.

The main symptom at admission was fever in 17/20 patients (85%), followed by irritative cough in 16 (80%), dyspnea in 15 (75%), fatigue in 14 (70%), and ageusia and/or anosmia in 2 patients (10%). Chest X-ray findings compatible with pneumonia were observed in 19 patients (95%), being multilobar in 18 (97.4%) and unilobar in 1 (5.3%). The radiological pattern was alveolar in three patients (15.8%), ground glass in seven (36.8%), and mixed in the remaining nine (47.4%). The main associated complication during hospitalization was respiratory distress in 14 (70%) patients; admission to the intensive care unit (ICU) was required for seven (35%) of these patients and invasive mechanical ventilation in five (25%). All patients were treated with corticosteroids, administered as a bolus in 14 patients (70%). Five patients (25%) received antiviral treatment and another six (30%) were treated with selective inhibitors of pro-inflammatory cytokines (five with tocilizumab and one with anakinra). Finally, three patients (15%) required home oxygen therapy at discharge.

### 3.1. The [^18^F]FDG-PET/CT Findings

**Figure 1:**
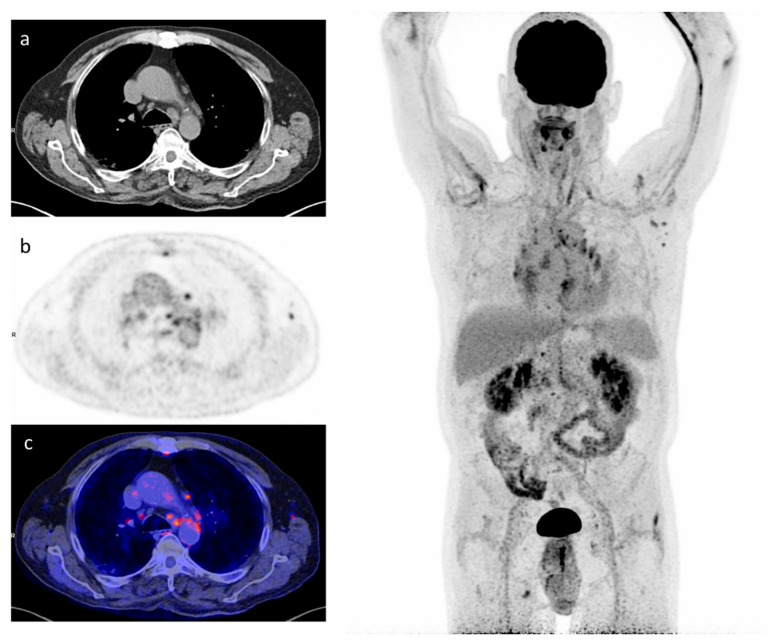
A 64-year man admitted for multilobar pneumonia caused by SARS-CoV-2. The [^18^F]FDG-PET/CT at 3 months after symptom onset shows increased [^18^F]FDG uptake in residual pulmonary lesions (TLG 124,11) and mediastinum lymph node (SUVpeak 1,73). Pulmonary function tests evidenced severe pulmonary diffusion impairment, with a diffusing capacity of the lungs for carbon monoxide 41% of the predicted value. Left: (**a**) CT transverse slice, (**b**) [^18^F]FDG-PET slice, and (**c**) fused [^18^F]FDG-PET and CT images. Right: whole-body, maximal intensity projection image, displaying mediastinal lymph nodes with [^18^F]FDG uptake.

The mean time from hospital discharge to [^18^F]FDG-PET/CT study was 58.85 ± 13.67 days. The result was positive in 11 patients (55%) and negative in 9 (45%). The main finding was hypermetabolic lymphadenopathy in the mediastinum, observed in 10 (90.9%) of the [^18^F]FDG-PET/CT-positive patients (Figure 1).

**Figure 2:**
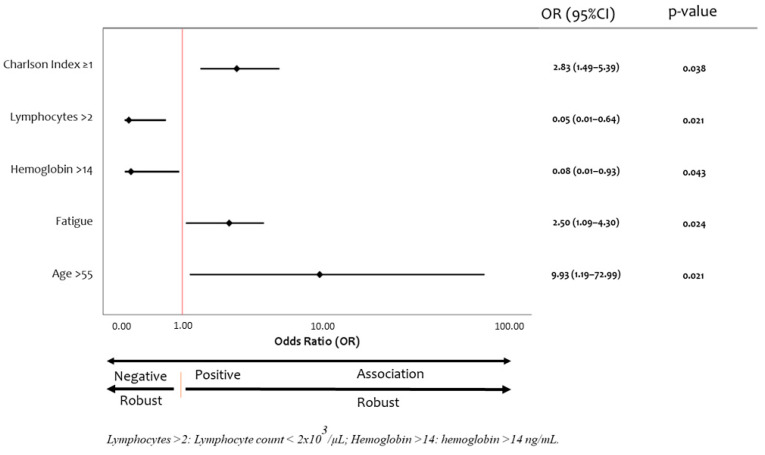
Factors associated with [^18^F]FDG-PET/CT positive. Forest plot with odds ratios shown by closed circles and 95% confidence intervals by whiskers.

Patients with positive and negative [^18^F]FDG-PET/CT results significantly differed in age (59.82 ± 8.52 vs. 51.00 ± 8.09 years, respectively, *p* = 0.03), Charlson index score ≥1 (66.7 vs. 100%, *p* = 0.038), presence of fatigue (90.9 vs. 44.4%, *p* = 0.024) and respiratory distress (90.9 vs. 44.4%, *p* = 0.024), hemoglobin levels (13.41 ± 1.91 vs. 15.24 ± 1.58 g/dL, *p* = 0.041), and lymphocyte count (1.78 ± 0.53 vs. 2.47 ± 0.49 × 10^3^/µL, *p* = 0.011) at 2–3 months post-discharge (Figure 2).

### 3.2. Correlation of Volumetric [^18^F]FDG-PET/CT Parameters with Laboratory Test Results

Table 2 exhibits associations observed between volumetric [^18^F]FDG-PET/CT results and analytical findings at admission, during the hospital stay, and at 2–3 months post-discharge.

At admission, a significant correlation was found between the SUVpeak of the target lesion in the mediastinum and the hemoglobin level (r = 0.615, *p* = 0.044), leukocyte count (rho = −0.664, *p* = 0.026), neutrophil count (rho = −0.764, *p* = 0.006), lymphocyte count (rho = 0.636, *p* = 0.035), and NLR (rho = −0.664, *p* = 0.026). In addition, the TLG in lung parenchyma was significantly correlated with C-reactive protein (CRP) (rho = 0.558, *p* = 0.011), procalcitonin (rho = 0.611, *p* = 0.035), fibrinogen (rho = 0.472, *p* = 0.041), and blood glucose (rho = 0.517, *p* = 0.020) levels at hospital admission.

During hospitalization, the SUVpeak of the target lesion was again significantly correlated with neutrophil count (rho = −0.700, *p* = 0.016), lymphocyte count (rho = 0.618, *p* = 0.043), and NLR (rho = −0.627, *p* = 0.039). Furthermore, pulmonary TLG was significantly correlated with IL-6 (rho = 0.624, *p* = 0.010), CRP (rho = 0.618, *p* = 0.004), procalcitonin (rho = 0.570, *p* = 0.042), LDH (rho = 0.445, *p* = 0.049), troponin (rho = 0.883, *p* = 0.002), fibrinogen (rho = 0.635, *p* = 0.015), and D-dimer (rho = 0.674, *p* = 0.001) levels and with the neutrophil count (rho = 0.615, *p* = 0.044), lymphocyte count (rho = −0.615, *p* = 0.004), and NLR (rho = 0.558, *p* = 0.011) during the hospital stay.

**Figure 3:**
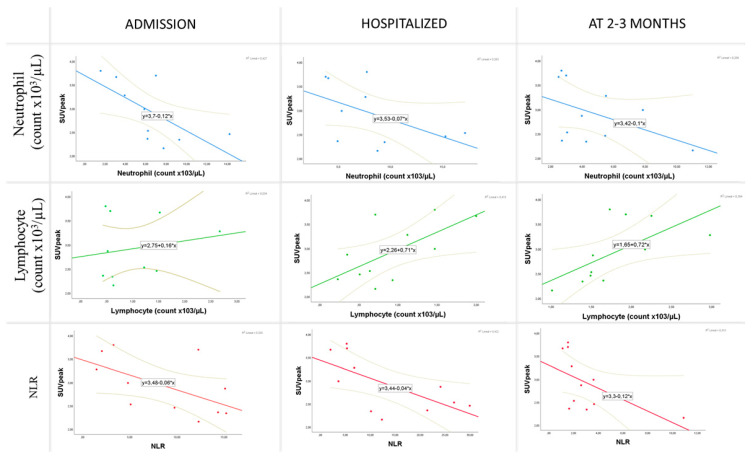
Scatter plot matrix. Correlation of the SUVpeak of the target lesion with neutrophil and lymphocyte counts and NLR at admission, during hospital stay, and at 2–3 months post-discharge.

At the follow-up at 2–3 months, the SUVpeak was significantly correlated with neutrophil count (rho = −0.679, *p* = 0.022), lymphocyte count (rho = 0.791, *p* = 0.004), and NLR (rho = −0.727, *p* = 0.011). No significant correlation was found between pulmonary TLG and any analytical parameter under study (Figure 3).

### 3.3. Correlation of Volumetric [^18^F]FDG-PET/CT Parameters with Respiratory Function Parameters

Eleven (55%) of the 20 patients had impaired respiratory function. Percentage predicted values were <80% for FVC in 20% of patients, <80% for FEV1 in 15%, <70% for FEV1/FVC in 5%, <80% for TLC in 20%, <80 for DLCO in 35%, <80% for KCO in 25%, and <65% for VR in 5%. Saturation was ≥4% lower at the finish versus start of the walk test in four patients (20%), and the distance was <400 m in three (15%).

**Figure 4:**
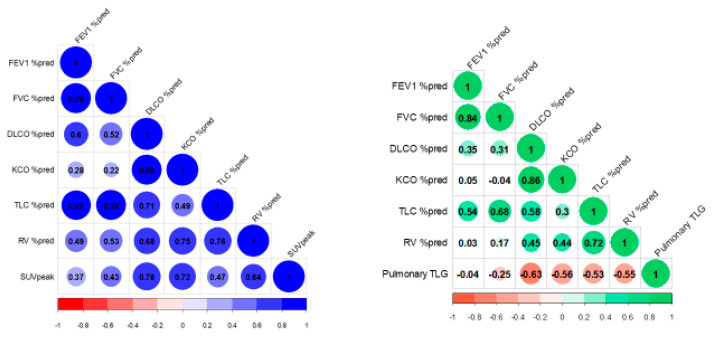
Correlogram showing the association of respiratory function test results with the SUVpeak of the target lesion and pulmonary TLG.

Volumetric [^18^F]FDG-PET/CT parameters were related to respiratory function test results obtained at 2–3 months post-discharge (Figure 4). The SUVpeak of the target lesion in the mediastinum was significantly and positively correlated with % predicted DLCO (rho = 0.782, *p* = 0.008), KCO (rho = 0.721, *p* = 0.019), and RV (rho = 0.636, *p* = 0.048) values. Pulmonary TLG was significantly and negatively correlated with % predicted DLCO (rho = −0.628, *p* = 0.005), KCO (rho = −0.564, *p* = 0.014), TLC (rho = −0.532, *p* = 0.023), and RV (rho = −0.554, *p* = 0.017) values. (Table 3).

## 4. Discussion

In this study, [^18^F]FDG-PET/CT was used to measure the metabolism of lungs and other organs in the short–medium follow-up of patients admitted to hospital for pneumonia or respiratory failure due to COVID-19 infection. Despite testing negative for the infection in two successive RT-PCR tests of nasopharyngeal swabs, more than half of the patients showed increased metabolic activity (i.e., persistent inflammation) on [^18^F]FDG-PET/CT images in lung tissue of normal appearance and in mediastinal lymph nodes. To our best knowledge, [^18^F]FDG-PET/CT has not previously been used to detect residual inflammatory processes after COVID-19 infection. These findings contribute evidence on the pathophysiological processes in patients who survive hospital admission for COVID-19 pneumonia.

The [^18^F]FDG-PET/CT has been employed in patients with influenza A, aspiration pneumonia, and organized pneumonia to assess the extent and severity of the disease, to follow its course, and to evaluate the response to therapy [5,18,19]. Research on the role of [^18^F]FDG-PET/CT in COVID-19 infection has generally focused on the acute phase. In this regard, Qin et al. reported high [^18^F]FDG uptake in lung lesions and mediastinal lymph nodes of four patients strongly suspected of the infection [4], and Colandrea et al. described elevated [^18^F]FDG uptake in lung lesions in 80% of a series of symptom-free oncology patients diagnosed with COVID-19 [20]. However, few studies have addressed the short- or medium-term consequences of COVID-19 infection. Dietz et al. recently reported increased [^18^F]FDG uptake in lung lesions and mediastinal lymph nodes of 13 non-critically ill COVID-19 patients at days 6–14 after symptom onset, although the short-axis diameter of mediastinal lymph nodes was always < 1 cm [3]. Johnson et al. proposed that high [^18^F]FDG uptake in mediastinal lymph nodes might be secondary to lung involvement in COVID-19 [21]. Bai et al. found elevated metabolic activity in residual lung lesions in COVID-19 survivors after two successive negative results in the RT-PCR test [22], and Scarlattei et al. reported that this metabolic activity remained high many weeks after the disappearance of symptoms and a negative RT-PCR test result [23]. The present results are in line with the above findings and contribute novel data on increased metabolic activity in lung tissue of normal appearance and in mediastinal lymph nodes of normal size. In this context, Xu et al. described lymphocyte-dominated interstitial mononuclear inflammatory infiltrates in both lungs of a patient with COVID-19 and reported that substantial inflammation may persist in the lungs after the disappearance of the infection [24]. The elevated [^18^F]FDG uptake would reflect increased glycolytic activity due to infiltration and inflammation of the lung, even in normally aerated areas that show no morphological alterations on CT images, demonstrating the greater capacity of [^18^F]FDG-PET/CT to detect inflamed lung areas in comparison to CT alone [8,22], which may persist long after the disappearance of COVID-19 infection. The possible duration of the post-COVID-19 inflammatory response in lungs and extrapulmonary sites has yet to be established and warrants further research.

At 2–3 months post-discharge, patients with elevated chest [^18^F]FDG uptake were older and characterized by a higher Charlson index, more frequent fatigue and respiratory distress, and lower hemoglobin and lymphocyte counts in comparison to those with normal [^18^F]FDG uptake. The SUVpeak of the target lesion and pulmonary TLG were significantly correlated with acute phase reactants and white blood cell counts at admission, during the hospital stay, and at 2–3 months post-discharge. Although there is a lack of similar studies in severely ill COVID-19 survivors for comparison with these results, they are consistent with previous findings on risk factors for more severe infection, including old age, underlying comorbidities [12,25], and similar changes in white blood cell counts, lymphocyte counts, procalcitonin and CRP levels, and NLR [26,27]. The [^18^F]FDG-PET/CT findings were correlated with the NLR in all studied phases of COVID-19 disease. The persistence over time of increased [^18^F]FDG uptake intensity may reflect a more severe acute phase of the disease.

The lung appears to be the most frequently involved organ in COVID-19, with reports of diffuse alveolar epithelium destruction, capillary damage/bleeding, hyaline membrane formation, alveolar septal fibrous proliferation, and/or pulmonary consolidation, among others [12,24]. Long-term follow-up studies of survivors of other coronavirus infections found that respiratory function limitations frequently last for months or even years, including impaired DLCO (in 15.5–43.6% of patients) and decreased TLC (5.2–10.9%) [28,29,30]. Various authors have addressed short- and medium-term respiratory function outcomes in survivors of COVID-19 infection, usually at hospital discharge [31,32]. In a study at 2–3 months post-discharge of 55 COVID-19 survivors who had not required mechanical ventilation, Zhao et al. described residual pulmonary function in 14 patients (25.45%), mainly impaired DLCO (in 13.6%) [33]. In a study at 6 weeks post-discharge of 124 COVID-19 survivors, van den Borst et al. [34] described an improvement in radiological images for almost all patients (99%) but observed residual lung parenchymal alterations in 91% of the patients and reduced lung diffusion capacity in 42%. Likewise, in their study at 3 months post-discharge of 76 healthcare workers who recovered from COVID-19, Liang et al. reported normal FEV1, FVC, FEV1/FVC, TLC, and DLCO values (>80% predicted) in 82% of the patients but the persistence of mild pulmonary function abnormalities in 42% [35]. The proportion of the present patients with impaired pulmonary function at 2–3 months was in line with previous findings on the short- to medium-term effects of COVID-19 infection [33,34].

The most frequent respiratory sequela of COVID-19 was DLCO alteration, as reported in previous studies, which may indicate the presence of pulmonary fibrosis [12,24]. DLCO and other respiratory function parameters were negatively correlated with the lung [^18^F]FDG uptake as quantified by TLG. Although only a small proportion of the present patients had severe airway dysfunction, the results suggest that COVID-19 produces diffuse pulmonary epithelial damage and mild congestion of the airway mediated by lymphocyte-dominated interstitial inflammatory infiltrates. No published data appear to be available on the association between respiratory function test results and pulmonary TLG. The majority of the present patients showed no lung lesions on CT scans at 2–3 months after discharge; however, pulmonary function was impaired in more than half of the patients with a normal lung CT scan. Hence, pulmonary function and [^18^F]FDG-PET/CT testing is more sensitive than CT alone for identifying candidates for pulmonary rehabilitation after SARS-CoV-2 pneumonia [22].

High [^18^F]FDG uptake may be related to increased anaerobic glycolysis caused by a cascade of reactions involving inflammatory cells [7,36]. In this way, the uptake of [^18^F]FDG by lung lesions and lymph nodes observed in this study may be due to nonspecific immune or inflammatory activation, similar to the high [^18^F]FDG uptake observed in lung lesions caused by the Middle East respiratory syndrome, pandemic H1N1 influenza virus, and organized pneumonia [18,19,37].

The [^18^F]FDG-PET/CT offers a complementary approach to other imaging modalities by providing metabolic information. Although not currently recommended for the diagnosis of COVID-19 in the acute phase [8], it can yield relevant information for the diagnosis of short- and medium-term complications, including the chronic damage to the lungs and extrapulmonary sites that can follow acute infection [6,22]. However, radiologists and nuclear physicians need to develop a thorough understanding of the cellular mechanisms that underlie the pathophysiology of COVID-19 in the clinical settings of lung and extrapulmonary malignancies and inflammatory diseases in order to avoid misinterpretation of [^18^F]FDG-PET/CT images [31].

Besides the small sample size, the main limitation of this study was the absence of a control group, hampering the possibility to detect causal relationships between the findings and COVID-19 infection. The epidemiological environment in which this study was carried out determined strict, restrictive conditions for access to hospital centers in our center and population. Evidently, the performance of [^18^F]PET/CT in healthy collaborating patients was obviously not authorized. In addition, no test results were available for the baseline respiratory function of patients before COVID-19, although the presence of chronic lung disease was an exclusion criterion. Further research is required to fully elucidate the impact of COVID-19 on pulmonary function. In this regard, the present results cannot be extrapolated to patients with chronic lung disease. Another study limitation was the absence of a follow-up period to explore the long-term clinical relevance of the respiratory function impairment. Finally, biopsy specimens were not available for the studied organs. Nevertheless, the present findings contribute to laying the foundations for future studies with larger series on the potential role of [^18^F]FDG-PET/CT in evaluating the sequelae of COVID-19 infection. These should have prolonged follow-up periods to explore the possible relationship between initial lung inflammation and long-term sequelae such as residual lung fibrosis and respiratory failure.

## 5. Conclusions

In conclusion, at 2–3 months after the acute phase of SARS-CoV-2 infection, almost half of the patients evidenced an impairment of pulmonary function that was correlated with [^18^F]FDG-PET/CT findings. In addition, the increased metabolic activity observed in the lung and mediastinal lymph node was associated with clinical and laboratory markers of disease severity. The [^18^F]FDG-PET/CT is useful to obtain novel information on the pathogenesis of COVID-19 and on the diagnostic and evaluation of short- and medium-term sequelae, contributing to their management.

## Figures and Tables

**Table 1:** Baseline clinical characteristics and risk factors of patients.

Clinical Characteristics (*n*)	Mean ± SD or *n* (%)
Age (years)	55.85 ± 9.28
Gender (Male)	12 (60)
BMI (kg/m^2^)	34.11 ± 7.23
**Comorbidities**	
Former or current smoking habit	2 (10)
Hypertension	5 (25)
Diabetes	3 (15)
Hyperlipidemia	2 (10)
Atrial fibrillation	2 (10)
Asthma	3 (15)
Charlson Comorbidity index	1.60 ± 1.14
Charlson Comorbidity index ≥ 2	9 (45)
**Clinical characteristics at admission**	
Fever	17 (85)
Dyspnea	15 (75)
Irritative cough	16 (80)
Fatigue	14 (70)
Myalgia	11 (55)
Anosmia/Ageusia	2 (10)
Digestive symptoms	9 (45)
Headache	3 (15)
ARDS (PaO_2_/FIO_2_ < 300 mmHg)	14 (70)
Blood oxygen saturation	90.90 ± 5.33
**Laboratory test results at admission**	
Hemoglobin (g/dL)	14.86 ± 1.84
White blood cell (count ×10^3^/µL)	7.68 ± 3.11
Neutrophil (count ×10^3^/µL)	6.28 ± 3.16
Lymphocyte (count ×10^3^/µL)	0.99 ± 0.57
NLR	8.36 ± 5.86
Platelet (count ×10^3^/µL)	206.65 ± 54.49
Ferritin (ng/mL)	1327.81 ± 1402.58
C-reactive protein (mg/L)	81.20 ± 54.61
LDH (U/L)	398.15 ± 113.80
AST(U/L)	49.25 ± 38.60
ALT(U/L)	48.30 ± 46.36
Albumin (g/dL)	3.92 ± 0.50
D-dimer (mg/L)	0.73 ± 0.52
**Characteristics of Hospitalization**	
Hospital stay (days)	16.70 ± 11.99
Pneumonia (chest X-ray)	19 (95)
ICU admission	10 (50)
Invasive mechanical ventilation	5 (25)
Bolus therapy with glucocorticoid	14 (60)
Antiviral therapy	5 (25)
Selective inhibitors of pro-inflammatory cytokines	6 (30)

Continuous variables are presented as means ± standard deviation (SD) and categorical variables as frequencies (percentages). ARDS: acute respiratory distress syndrome; AST: aspartate aminotransferase; ALT: alanine transaminase; NLR: neutrophil/lymphocyte ratio; PCT: procalcitonin; NT-proBNP: N terminal pro-B-type natriuretic peptide; LDH: lactate dehydrogenase; ICU: intensive care unit.

**Table 2:** Bivariate correlations of volumetric [^18^F]FDG PET/CT parameters with laboratory parameters at admission, during hospital stay, and at 2–3 months post-discharge (short-term follow-up).

Variable		SUVPeak		Pulmonary TLG	
		Spearman’s rho	*p*-Value	Spearman’s rho	*p*-Value
**Admission**	Hemoglobin (g/dL)	−0.664	0.026		
	Neutrophil count	−0.764	0.006		
	Lymphocyte count	0.636	0.035		
	NLR	−0.664	0.026		
**Hospital stay**	Neutrophil count	−0.700	0.016		
	Lymphocyte count	0.618	0.043		
	NLR	−0.627	0.039		
	IL-6			0.624	0.010
	C-reactive protein			0.618	0.004
	PCT			0.570	0.049
	LDH			0.445	0.049
	Troponin			0.883	0.002
	Fibrinogen			0.635	0.015
	D-dimer			0.674	0.001
**Short-term follow-up**	Neutrophil count	−0.679	0.022		
	Lymphocyte count	0.791	0.004		
	NLR	−0.727	0.011		

IL-6: interleukin 6; LDH: lactate dehydrogenase; NLR: neutrophil/lymphocyte ratio; PCT: procalcitonin.

**Table 3:** Bivariate correlations of volumetric [^18^F]FDG PET/CT parameters and respiratory function parameters in the short-term follow-up.

Variable	SUVpeak		Pulmonary TLG	
	Spearman’s rho	*p*-Value	Spearman’s rho	*p*-Value
**DLCO% pred**	0.782	0.008	−0.628	0.005
**KCO% pred**	0.721	0.019	−0.564	0.014
**TLC% pred**	0.467	0.174	−0.532	0.023
**RV% pred**	0.636	0.048	−0.554	0.017

DLCO: diffusing capacity of the lungs for carbon monoxide; KCO: CO transfer coefficient; RV: residual volume; TLC: total lung capacity.
